# Strength, Hardening, and Failure Observed by In Situ TEM Tensile Testing**

**DOI:** 10.1002/adem.201200031

**Published:** 2012-05-07

**Authors:** Daniel Kiener, Petra Kaufmann, Andrew M. Minor

**Affiliations:** 1 Department of Materials Physics, Montanuniversität Leoben, Jahnstraße 128700 Leoben, Austria; 2 National Center for Electron Microscopy, Lawrence Berkeley National Laboratory94720 Berkeley, CA, USA; 3 Department of Materials Science and Engineering, University of California94720 Berkeley, CA, USA

## Abstract

We present in situ transmission electron microscope tensile tests on focused ion beam fabricated single and multiple slip oriented Cu tensile samples with thicknesses in the range of 100–200 nm. Both crystal orientations fail by localized shear. While failure occurs after a few percent plastic strain and limited hardening in the single slip case, the multiple slip samples exhibit extended homogenous deformation and necking due to the activation of multiple dislocation sources in conjunction with significant hardening. The hardening behavior at 1% plastic strain is even more pronounced compared to compression samples of the same orientation due to the absence of sample taper and the interface to the compression platen. Moreover, we show for the first time that the strain rate sensitivity of such FIB prepared samples is an order of magnitude higher than that of bulk Cu.

Small volumes exhibit mechanical properties remarkably different from bulk materials.^1^ While there are several consistent trends such as an inverse scaling of strength with sample size,^2^–^5^ there is still considerable discussion on the underlying strengthening mechanisms. Focusing on face centered cubic (fcc) metals, simulations suggested that the increased strength can be explained by truncated spiral dislocation sources,^6^–^8^ as was confirmed by a number of in situ transmission electron microscopy (TEM) experiments.^9^–^12^ A different approach suggests that the strength results from a lack of dislocations in the sample,^13^ again supported by simulations^14^ and experimental evidence.^15^

In body centered cubic (bcc) metals, a different small-scale deformation behavior as compared to fcc metals has been proposed.^16^ Molecular dynamics simulations have shown that deformation of small bcc volumes can lead to a loss of dislocations at low strengths, but actually lead to increased dislocation densities at high strengths,^17^ a hypothesis supported by recent in situ TEM results.^18^

Furthermore, it was pointed out that even the sample fabrication can play a significant role on the observed results.^19^ The most frequently used fabrication technique for such samples is focused ion beam (FIB) milling.^1^ There are alternative fabrication techniques such as etching,^20^ directional solidification,^21^ imprinting,^22^ or electroplating.^23^ However, all methods have tradeoffs in terms of material flexibility, microstructure control, surface roughness, site selectivity, and sample geometry. Thus, due to its versatility FIB fabrication in general remains the most common technique in the field. The main drawback of this technique is that it creates defects in the near surface region of the sample.^24–27^ It was shown that such defects drop the strength of pristine bcc Mo alloy whiskers from close to the theoretical strength^21^ to that observed for FIB fabricated ones^19^ or heavily pre-deformed material.^28^ Reports on similar effects also exist for fcc Au.^29^ It was, however, recently shown that this damage effect can be removed by thermal annealing of the sample for bcc Mo^30^ and fcc Cu,^31^ respectively. Moreover, for studying materials containing high obstacle densities, no significant influence of the FIB damage is observed.^10^, ^19^, ^25^, ^29^, ^32^, ^33^

Another complication arises from the sample tapering typically observed during FIB fabrication when using annular milling.^24^, ^26^ While on the micron scale there are techniques to avoid this problem,^1^, ^34^, ^35^ many groups prefer the simpler annular milling approach, and with the current instrumentation it is hard to envision 80 nm samples^36^ fabricated taper free by a more sophisticated approach.^1^ As a consequence, the sample taper can significantly influence the results of annular milled micro- and nanopillars. For example, a study on a metallic glass showed that an apparent sample size effect on strength disappeared once geometric inaccuracies were taken into account,^37^ as confirmed recently for a number of different metallic glasses.^38^, ^39^

Using in situ TEM, it is possible to determine the actual contact area and thus true stresses for such tapered nanoscale samples.^36^ However, there remains the question of whether the mechanical response of a tapered sub-micron pillar that deforms locally at the sample top due to stress concentrations^36^ is comparable to that of a larger sample volume with the same minimal dimension.^40^

In order to address the shortcoming of small-scale compression testing, we recently developed a quantitative in situ TEM tensile approach.^11^ This technique allows to probe the properties of non-tapered tensile samples, thereby sampling a significantly larger volume as compared to compression tests, and remove the influence of the flat punch interface (see Supporting Information). It was shown previously that the yield strengths are in good agreement between tension and compression when mechanical data is derived based on the true sample dimensions at the first dislocation burst event.^11^ However, comparing the complete deformation behavior as opposed to simply the yield strength reveals differences in tension and compression testing at this scale. For instance, extended homogenous deformation was observed for multiple slip oriented tensile specimens, but not for compression specimens of the same material.^36^ It is the aim of this work to examine the flow and hardening characteristics as well as the failure behavior of sub-micron tensile tests in comparison to compression tests of tapered pillars from the same Cu single crystal. Moreover, for the first time we demonstrate significant strain rate effects for a FIB fabricated fcc material at sub-micron length scales.

The yield behavior in Cu is not particularly sensitive to the crystal orientation of the specimen, since Cu is an elastically rather isotropic material^41^, ^42^ and the main controlling aspect is the location of the weakest source.^6^, ^12^, ^43^ The situation is presumably different for the subsequent hardening behavior, as the probabilities for dislocation interaction depend significantly on the slip geometry.^44^ Thus, it is necessary to compare samples oriented for single or multiple slip, respectively.

The true stress versus true strain data for in situ TEM tensile tests for multiple slip and single slip oriented samples is shown in Figure 1. The two tests in Figure 1a had a comparable thickness of 132 and 161 nm and were both oriented for multiple slip with a [100] pulling direction. The thinner sample (gray curve) was fully unloaded after straining to 3% plastic strain, while the thicker one was strained in a single experiment. In both cases, the negative loads after elastic unloading stem from adhesion between sample head and diamond gripper. It is seen that the two samples yield at stresses of ≍650 and ≍675 MPa and exhibit pronounced hardening in a similar manner. The full unloading seems not to significantly alter the deformation behavior. Figure 1b presents two single slip oriented specimens. The gray curve corresponds to a sample with a thickness of 168 nm, comparable to the tensile tests shown in Figure 1a, and yields at a similar stress of ≍690 MPa. The black data corresponds to a thicker sample (273 nm) that yielded at a lower stress of ≍570 MPa. The highest Schmid factors for <1−10>{111} slip in the (100) and (234) oriented samples are 0.408 and 0.422, respectively. Thus, the difference between the two orientations from converting the normal stresses into shear stresses would be ≍3.5%, which is negligible compared to the typical scatter observed for such small-scale tests.^1–5^, ^45–47^

**Fig 1:**
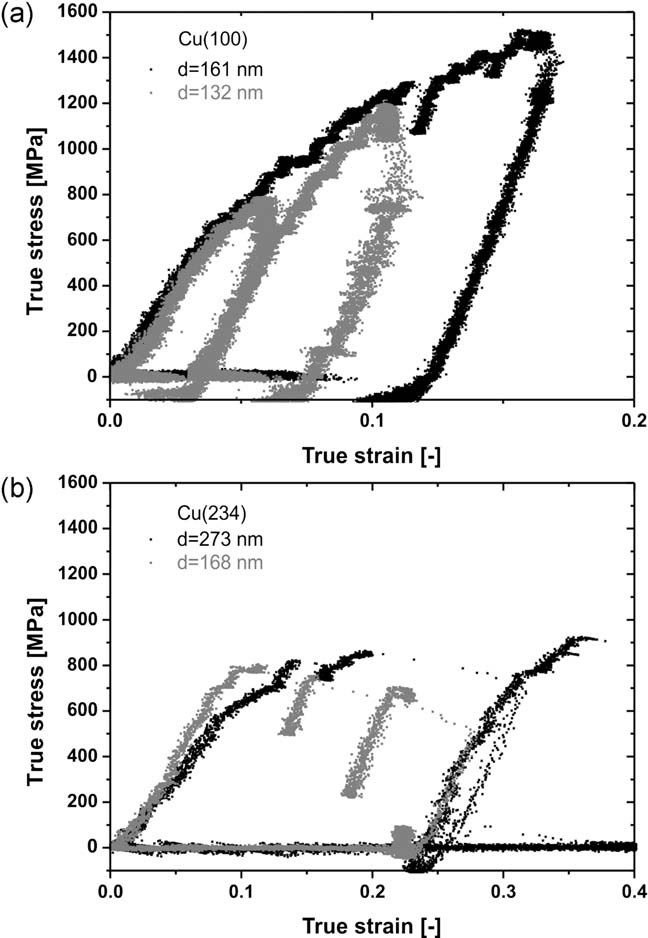
True stress versus true strain data from in situ TEM tensile tests on Cu samples oriented for multiple slip (a) and single slip (b). The sample thicknesses are given in the graphs.

The different hardening behavior between the single slip and multiple slip orientation samples (Figure 1) can be understood by considering the in situ observations. In the still images extracted from two subsequent loadings of a multi-slip tensile specimen (gray curve in Figure 1a) at least two different spiral dislocation sources are observed after yield, operating at different positions in the sample at increasing stresses as indicated by arrows (Figure 2a–d). From the different propagation directions of the dislocations emitted from the two sources, we assume that they glide on different slip systems. Instead of strain localization, homogenous elongation along with an according reduction in width was observed. This behavior is observed for (100) oriented Cu up to several 10% true strain.^11^ Moreover, this is in agreement with a straining experiment on a (100) oriented Al specimen having a diameter of ≍455 nm,^9^ where alternating operation of multiple spiral sources was observed at a strain rate of ≍1 × 10^−4^ s^−1^ up to a strain of ≍160%. Contrary, surface mediated dislocation nucleation would occur at significantly higher stress levels (requiring several per cent elastic strain),^48^ while in Figure 1 only ≍1% elastic strain was reached.

**Fig 2:**
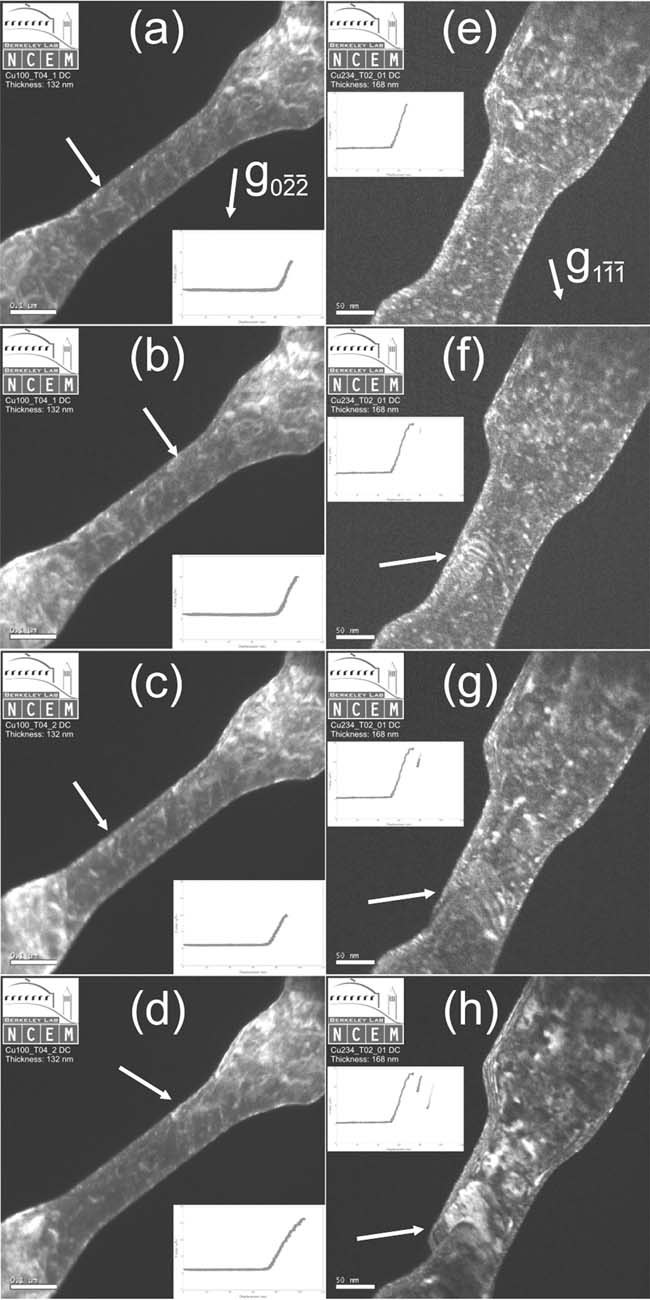
In situ images from TEM tensile tests on Cu samples oriented for multiple slip (a–d) and single slip (e–h). In the multiple slip case, the alternating operation of different sources (arrows) occurs at increasing stresses. Contrary, in the single slip sample the operation of a single spiral dislocation source leads to localized deformation (arrows) without pronounced hardening. The imaging conditions are indicated in (a) and (e).

A series of still images from the test shown in Figure 1b (gray line) are shown in Figure 2e–h. The point immediately before yield is depicted in Figure 2e. As indicated by the arrows, during plastic deformation (Figure 2f–h) only a single dislocation source was observed to operate. This resulted in the immediate formation of a slip step, where further plastic deformation localized in the absence of hardening. Occasionally, some initial hardening in the low strain regime was observed even in single slip oriented samples. From the in situ observations, this was ascribed to the consecutive operation of two spiral sources in a tensile sample (when the sources were only seen to have a single pinning point we assume these are spiral sources). Once dislocations emitted from the weakest source get blocked, for example, by sessile dislocations,^11^, ^36^ the source is shut down by the exerted back stress or changes of the local stress state, and the second weakest spiral source starts to operate at higher stresses. Once only a single source prevailed in the specimen, slip localization followed as shown in Figure 2e–h.

Interaction of dislocations emitted from different sources in order to form new pinning points for spiral sources is not expected in the single slip orientation, since for this geometry the dislocations glide on remote slip planes that are presumably parallel to each other.

Contrary to the tensile results, during nanocompression testing of multi-slip oriented Cu samples (fabricated on the same single crystal wedge as the tensile specimens), localized deformation was always observed for sizes below ≍200 nm.^36^ This presumably resulted from the limited volume experiencing the highest stresses during compression of a tapered pillar in conjunction with friction at the interface to the diamond flat punch. The larger sample volume loaded in a tensile test removes such limitations and allows for a clearer analysis of the deformation behavior.

The failure behavior of miniaturized systems is of immanent importance, but not addressable by compressive loading. Similar to macro-scale tests, tensile loading is the preferred method to address failure in metals. For single slip oriented samples (Figure 2e–h) the failure behavior is a clear result of the localized deformation. Once such a shear offset is formed, the local stresses increase by the reduction in cross-sectional area, which in turn drives final failure by shear at low to moderate strain. In Figure 3, still images from a loading sequence of an ≍105 nm thick multi-slip sample are shown. This specimen was already elongated to 25% total plastic strain, and operation of several spiral sources was observed (not shown here). Resultant to this deformation, the specimen shows a significant amount of necking, indicated by dashed lines in Figure 3a. Few video frames later (Figure 3b), the sample is completely pulled apart in a very fast event. The specimen seems to be still intact, as also seen in Figure 3c, but was in fact ripped apart and then put back in place by the displacement feedback control within several milliseconds, too fast to capture on video but evident from the load–displacement data. After the system was back to the prescribed displacement, it slowly pulled the specimen apart again at a decreasing low load (Figure 3d), which presumably emerges from adhesion between the newly exposed surfaces of the two sample parts.

**Fig 3:**
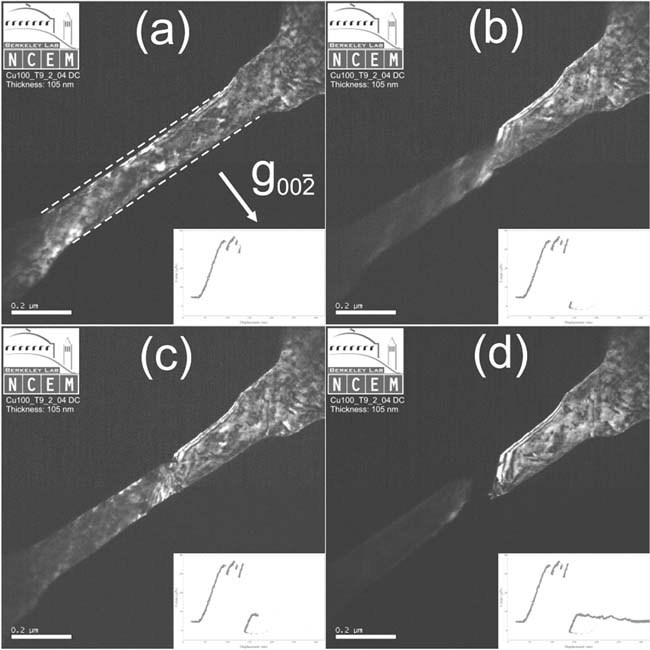
Still images from an in situ TEM tensile test of a 105 nm thick Cu sample oriented for multiple slip. (a) Operation of several different sources during the previous straining to 25% plastic strain (not shown here) led to sample necking. (b) A dislocation source is activated and produces a large slip offset along which the sample fails rapidly. (c) The feedback for the displacement control puts the sample parts together again. (d) Subsequently, the specimen is further pulled apart at the programmed strain rate.

The final failure mechanism is for both orientations localized shear resultant to operation of a single spiral source. The main difference is that this happens at rather low plastic strain for single slip oriented samples, since they lack strengthening mechanisms to compensate for the geometrical softening. In the case of multiple slip specimens, the localized failure occurs at much higher plastic strains and is preceded by hardening and necking due to the operation of multiple spiral sources.

It should be noted that when the sample fails, due to the rapid softening the internal feedback control (though working internally with 79 kHz^49^) overshot the prescribed displacement by ≍150 nm (Figure 3b), during which the sample was sheared off. Subsequently, the control mechanism moved the tip back to the prescribed position. This happens within several milliseconds, thus within a single video frame, which allows us here to only show the fracture site after the sample was finally pulled apart again.

Such localized deformation and shear failure was previously reported during in situ SEM tensile testing of defect free (111) oriented Cu whiskers in a similar size range.^50^ For such dislocation free samples, nucleation of the first dislocation is expected from the surface. This process can immediately lead to shear localization, since nucleation of the next dislocation could occur at lower stresses due to the presence of a surface step.^48^ More recently, a study on sub-micron Al fibers reported shear failure for samples with a low dislocation density of ≍5 × 10^11^ m^−2^, while higher dislocation densities resulted in necking before failure.^12^ Our studies confirm these previous reports, but also extend them by considering the distinct influence of the crystal orientation on the plastic behavior in sub-micron dimensions.

A different behavior was reported in another in situ SEM study on electro-plated (111) oriented Cu pillars, where extensive necking from the onset of plastic deformation close to one sample end was reported.^51^ Possible reasons for this might be the surface roughness of ±2 nm of these electro-plated structures facilitating surface nucleation, whereas physical vapor deposited^50^ or FIB prepared specimens have a rather flat surface. Also, due to the sample fixation using metal deposition for pulling the electro-plated pillars, a taper evolved and only the top part of the specimens deformed.^51^ The situation is further complicated by the occasional observation of twins within some electro-plated samples.^51^ Thus, no detailed comparison of the electro-plated pillars to the present results is attempted.

Having described the deformation, hardening, and failure mechanism of these nanotensile tests, we will next discuss the size dependent hardening behavior of single and multiple slip tensile and compression samples. The hardening data of the tensile samples was evaluated by the method suggested by Guruprasad and Benzerga.^52^ The same approach was previously used for the nanocompression tests^36^ to facilitate comparison, and the resultant data is presented in Figure 4. It was shown that the nanocompression data exhibits size- and strain-dependent hardening behavior, with higher hardening rates measured for smaller samples and lower amounts of plastic strain. For comparison, the bulk data for stage II hardening of Cu single crystals is indicated by a shaded regime.^53^, ^54^ The fact that the data points for the compression tests shift to larger diameters upon deformation reflects the effect of sample taper, which increases the plastically deforming diameter with ongoing compression. This effect is far less pronounced for the tensile data, as these samples were taper free and thus should only reduce their dimensions according to Poisson's contraction and thinning due to plasticity. The reduction in diameter with strain is particularly noticeable for multiple slip specimens, as they showed significant necking. For the single slip samples, the reduction in diameter was essentially negligible since they did not show significant necking. It should be pointed out that a determination of true stresses and strains was not attempted once localized sample deformation had occurred (e.g., Figure 2h and 3b). The reason we did not attempt this analysis after localization was because, depending on the Burger's vector of the dislocations producing the slip step, the out-of-plane slip offset can be significantly different from the visible in-plane component, leading to errors in determining the cross-section.

**Fig 4:**
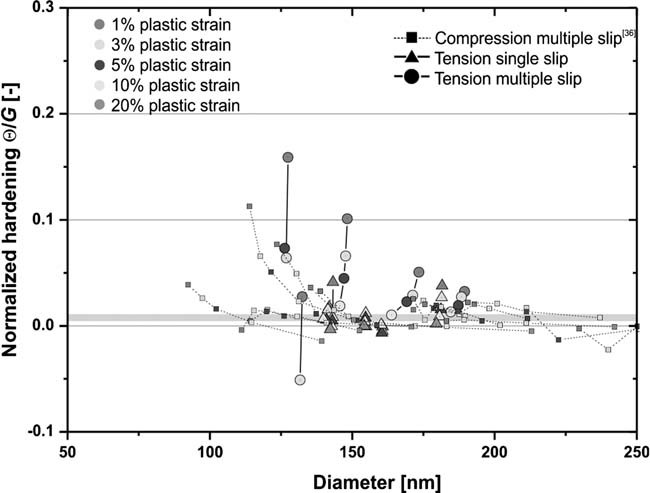
Comparison of the normalized hardening rate Θ/*G* for compression^36^ and tensile tests as function of plastic strain and size. Although sampling a larger volume, the tensile tests exhibit higher hardening rates than the compression experiments at low plastic strain. The shaded area depicts the bulk hardening values for stage II hardening of Cu.

The limited hardening behavior of the single slip oriented samples (triangle symbols in Figure 4) is easily explained by the immediate strain localization (Figure 2h and 3b). The more important observation is that the multiple slip tensile samples show consistently higher hardening rates than the compression samples for low plastic strains of 1%, while it was mentioned previously that the yield strengths are in close agreement.^36^ This discrepancy in hardening is surprising, as the larger homogenously stressed volume of a tensile sample should for statistical reasons contain more suitable dislocation sources to carry plastic deformation. A significant difference between tensile and compression tests, however, is the interface present between the compression sample and diamond tip. Besides the highest stresses being localized at this interface due to pillar taper, it was also shown that friction creates a multi-axial stress state in this area.^55^, ^56^ It appears that these effects facilitate the operation of dislocation sources near the interface and subsequent formation of slip steps. Once surface steps are formed, they themselves can promote subsequent nearby dislocation nucleation or source activation by the stress concentration they cause at the sample surface.^48^ This would lower the observed hardening rates in compression as compared to tension, and indeed no pronounced slip steps are observed at low plastic strain during tensile testing. While this provides a rationale for the observed differences, it also questions whether the values determined in compression at such small scales are in fact an intrinsic material property, or rather a result influenced by the testing configuration consisting of tapered pillar and flat punch in the given compression geometry.

Finally, motivated by earlier reports on strain rate influences,^9^ we investigate the effect of an increased strain rate on the stress versus strain behavior of nanoscale tensile samples by a strain rate jump experiment. An initially 132 nm thick Cu sample oriented for multiple slip was consecutively strained in four tests to a total plastic strain of 32.4% at a low strain rate of 5 × 10^−3^ s^−1^, see the true stress versus true strain data (black data points) in Figure 5. As previously shown for other samples, the specimen exhibits significant hardening without strain localization. Then, to perform the high strain rate test (gray data points), the loading protocol was slightly changed. The sample was first loaded and unloaded in the elastic regime only at the low strain rate, after which the specimen was immediately reloaded with a high strain rate of 1.8 s^−1^. The reason for this additional low strain rate step before the high strain rate loading was to confirm proper alignment, since the latter test would happen too fast for any imaging, and any additional bending from misalignment might lower the flow stress. The slopes of the last low strain rate test, that of the low rate alignment check, and the actual high rate test are all in good agreement, as can be seen in Figure 5, confirming proper alignment (the low strain rate alignment check is the thick gray line beginning at 32.4%).

**Fig 5:**
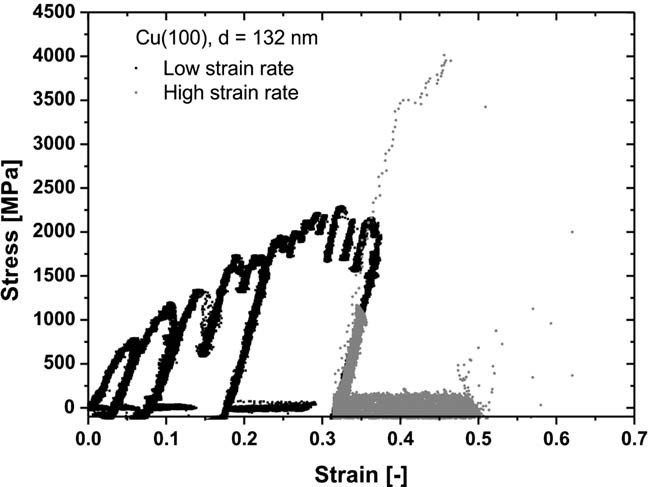
Stress versus strain tensile data for a multiple slip oriented 132 nm thick specimen. After consecutive straining to 32.4% plastic strain at a strain rate of 5 × 10^−3^ s^−1^ (black data points), the strain rate was increased to 1.8 s^−1^ (gray data points). This resulted in a dramatic increase of the flow stress before failure occurred at 4 GPa.

The striking effect of the increased strain rate can be clearly seen in Figure 5. While during the low strain rate test true flow stresses between 2 and 2.3 GPa were achieved, the sample starts to plastically deform in the high strain rate test at ≍3.5 GPa and finally fails at stresses exceeding 4 GPa. It should be pointed out that the high strain rate data is technically stress versus strain only, as only two blurred images were captured during this part of the test that took ≍60 ms, and conversion to true stress versus true strain was not attempted from these images. The failure behavior was comparable to previous results (Figure 3 and Supporting Information), the two parts of the specimen separated by shear in the middle of the sample. Such extraordinarily high stresses were previously only measured for pristine Cu whiskers^50^ and match the lower bound value for the theoretical strength of Cu as derived from ab initio calculations.^57^

Molecular dynamics simulations predicted strain rate sensitivity, even for fcc materials, for dimensions in the range of several tens of nm.^58^ This is explained by the thermal contribution to the surface nucleation of dislocations at such small scales and high stresses, which is negligible in the micron regime.^59^ While strain rate sensitivity for micron and sub-micron bcc pillars was experimentally observed earlier,^46^ it was only recently that a study on sub-micron electro-plated Cu pillars reported experimental evidence for strain rate sensitivity of sub-micron fcc samples, but at the same time noted the complete lack on such information for FIB fabricated structures.^60^ For the electro-plated Cu the authors reported a rather high strain rate sensitivity *m* between ≍0.027 and ≍0.057 at 10% strain, which was attributed to spiral dislocation source operation. Notably, those values are significantly larger than the range of 0.004–0.0072 reported for coarse grained bulk Cu.^61^ Moreover, for pillars in the range of 125–75 nm, the authors observed an increase in *m* to ≍0.11 for strain rates lower than 10^−1^ s^−1^. This was interpreted to arise from the highly thermal nature of surface dislocation sources.^60^

While our true stress of 1120 MPa at 10% strain for a strain rate of 5 × 10^−3^ s^−1^ agrees well with the electro-plated data,^60^ our strain rate jump test cannot be directly compared to these results, as our strain level and accordingly the stresses are much higher. We evaluate the strain rate sensitivity by taking the flow stress of 2.1 GPa at the end of the slow strain rate test and the “yield” stress of 3.5 GPa at the high strain rate, using the fact that the plastic strain is the same then. This gives a value of *m* = 0.08, which is an order of magnitude above the bulk data,^61^ and also higher than the values of ≍0.027 to ≍0.057 in the high strain rate regime of the electro-plated pillars.^60^ Still, the value is below what was attributed to be surface dislocation nucleation dominated behavior (*m* ≍ 0.11).

The reason for these differences could be that the previously mentioned surface roughness of the electro-plated pillars in conjunction with the interface to the diamond tip facilitates surface nucleation. In our results, the tensile approach removes the interface issue and the FIB defects in the surface region of the tensile sample provide short dislocation segments or loops that could evolve into spiral sources at such high stresses, thereby promoting spiral source operation. For a rough lower bound estimate, we assume dislocation segments from FIB preparation with a length of ≍5–10 nm, which is the approximate penetration depth for 30 keV Ga^+^ ions in Cu under grazing impact.^25^ The activation stress for a spiral source of this size range would be ≍1–1.5 GPa, which is within the range of flow stresses observed in our experiments. This points to the ubiquitous importance of the surface structure in such nanoscale dimensions, but a more detailed study is required for a thorough discussion of this effect, which is beyond the scope of this work.

In summary, we presented in situ TEM tensile experiments on specimens with the thinnest dimensions in the range of ≍100 to ≍200 nm and with crystal orientations to promote single slip or multiple slip, respectively. The single slip oriented samples exhibit only limited homogenous deformation before they fail by slip localization, while samples oriented for multiple slip show extensive homogenous deformation to high plastic strains and necking before failure by shear occurs. The hardening rates at 1% plastic strain in tension are even higher than what was observed for nanocompression tests, even though a larger volume is sampled during tensile testing. This is ascribed to the taper of the compression samples in conjunction with the interface between compression sample and flat punch that both promote localization of dislocation nucleation and deformation, thereby lowering the hardening rate. Finally, for the first time a pronounced strain rate effect was quantified for FIB-fabricated sub-micron fcc samples. The high strain rates resulted in stresses approaching the ideal strength and a strain rate sensitivity even higher than what was observed for electro-plated Cu pillars in compression.

## 1. Experimental

The experimental details with respect to sample preparation, in situ loading in the TEM and data evaluation were published previously 11, 36. Since the systematic comparison between tension and compression experiments is the scope of this work, only a brief summary of the experimental technique will be provided here. For full details please refer to the indicated publications.

### 1.1. Sample Preparation

Melt grown Cu single crystals with a purity of 99.999% and oriented for single slip (234) and multiple slip (100) were obtained (MaTecK GmbH, Juelich, Germany). From these crystals thin slices were cut using a diamond wire saw, subsequently grinded and finally electrochemically etched to a wedge shape. This wedge was fixed to a TEM sample support compatible with a PI 95 Picoindenter (Hysitron Inc., Minnesota, MN, USA), operated using a Perfomech controller. A FIB (FEI Strata 235; FEI, Hillsboro, OR, USA) working with Ga ions at 30 keV and a final milling current of 10 pA was used to mill tapered pillars with a tapering angle of 2.5° by the annular milling technique and non-tapered tensile samples as introduced in ref. 11. The compression samples has high aspect ratios between diameter and length of 1:5 to minimize for boundary constraints 56. The tensile samples generally had a 1:5 aspect ratio, sometimes with a dimensionally slightly reduced gauge section in the middle to ease detailed observation during loading.

### 1.2. In Situ Testing

In situ compression and tensile tests were conducted in a JEOL 3010 (JEOL, Tokyo, Japan) TEM working at 300 keV accelerating voltage. The tests were carried out in displacement controlled mode, applying strain rates in the range of 3–5 × 10^−3^ s^−1^ if not stated differently. For the compression tests, a conducting diamond tip with a 2 µm diameter flat end was used, while for the tensile tests a conductive diamond tip was FIB structured to a custom tensile gripper 11, 62. During the tests, load and displacement were recorded with 1 kHz, and the typical noise level without contact between sample and tip was ±1 µN and ±5 nm, respectively. In addition, a video of the experiments was recorded at 30 frames per second using a Gatan Orius SC200D (Gatan, Pleasanton, CA, USA) CCD camera.

### 1.3. Data Evaluation

By correlation between the actual sample dimensions from the in situ video and the load versus displacement data, the true stress versus true strain response was derived for all samples 36. This data was also used for later evaluation of the hardening rates. Here, we followed the approach of Guruprasad and Benzerga 52 as previously used for the nanocompression tests 36 to ensure compatibility of the data. In brief, the normalized hardening rate for a given plastic strain is calculated by a tangential approach for the stress increment between the yield point and the flow stress at the specified plastic strain.

## Notes

Detailed facts of importance to specialist readers are published as ”Supporting Information”. Such documents are peer-reviewed, but not copy-edited or typeset. They are made available as submitted by the authors.
