# Supplementary material for: Strength, Hardening, and Failure Observed by In Situ TEM Tensile Testing
Source: Adv Eng Mater. 2012 May 7;14(11):960–7. doi: 10.1002/adem.201200031 (PMC3573867; doi:10.1002/adem.201200031)
Supplement: Supplementary file 1 [file adem0014-0960-SD1.pdf]

DOI: 10.1002/adem.201200031

Strength, hardening and failure observed by in situ TEM tensile testing**

By *Daniel Kiener**, *Petra Kaufmann*, and *Andrew M. Minor*

Supporting online information

The Supplementary Figure 1 resembles a higher resolution image of the table of content figure, showing a bright field TEM image of a fractured single slip Cu tensile sample. The top left inset is the corresponding load – displacement data, while the bottom right image shows the right half of the fractured sample using a g_{022} dark field imaging condition.

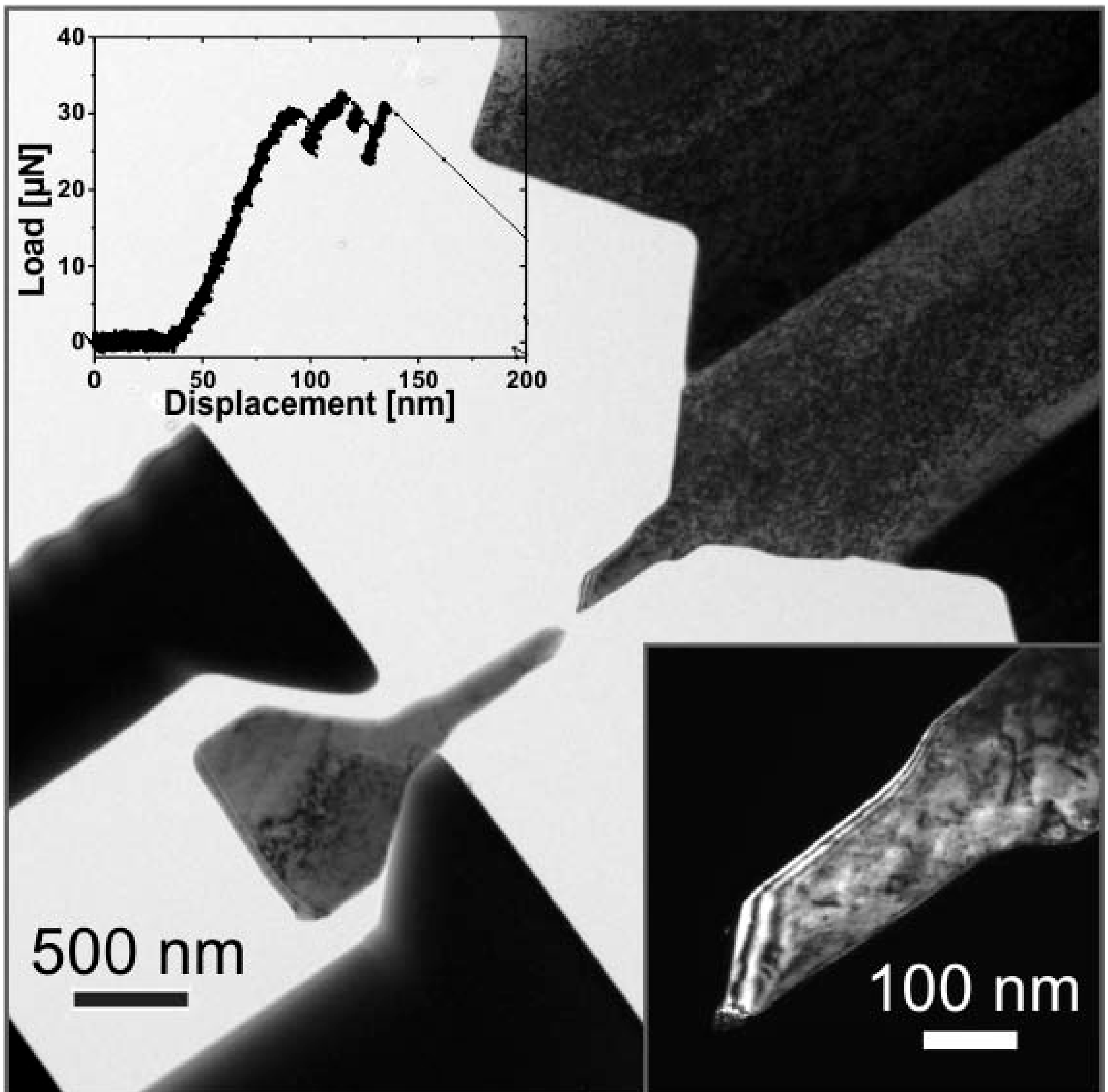

Supplementary Figure 1. Higher resolution image of the TOC figure, showing TEM images of a fractured single slip Cu tensile sample.
